# Conceptions of bibliographic managers in university teachers. An approach in Latin American

**DOI:** 10.12688/f1000research.143691.1

**Published:** 2024-03-21

**Authors:** Irene Roxana Abad-Lezama, Nathalí Pantigoso-Leython, Félix José Colina-Ysea, Gleny Secibel Jara-Llanos

**Affiliations:** 1 Academic Department of History and Geography, Universidad Nacional de Educacion Enrique Guzman y Valle, Lurigancho-Chosica, Lima, 15472, Peru; 2 Academic Department of Foreign Languages, Universidad Nacional de Educacion Enrique Guzman y Valle, Lima District, Lima Region, 15472, Peru; 3 Academic Department of Pedagogical Sciences, Universidad Nacional Experimental Francisco de Miranda, Falcón, Venezuela; 4 Academic Department of Communication and Native Languages, Universidad Nacional de Educación Enrique Guzmán y Valle, Lurigancho-Chosica, Lima, 15472, Peru

**Keywords:** Bibliographic managers, information organization, scientific sources, research

## Abstract

**Background:**

Due to the accumulation of information generated by the information society, it is necessary to establish scientific criteria for its systematization. The objective of the research was to understand the implicit theories on the use of bibliographic managers in the organization of scientific information in university teachers in Latin American university teachers (Colombia, Ecuador and Peru).

**Methods:**

The interpretative paradigm, phenomenological method and qualitative approach were used. The key informants were defined by fifteen teachers with research experience. The technique was the semi-structured interview, and the instrument was the interview script. The study was based on four cyclical-recursive moments, the first was the natural attitude, where information was obtained from the key informants as a result of the interviews. The second moment allowed the epoche-eidetic reduction, consisting of the analysis of each interview. The third moment originated the transcendental epoche-reduction, analyzing each attribute and their consistency. The fourth moment revealed the constitution of the study, integrating in a semantic network the categories, subcategories, and attributes that emerged.

**Results:**

Even when it is recognized from the cognitive conception about the concept of bibliographic managers, its applicability or use, it seems not to be so effective in scientific academic spaces and activities, because teachers are still anchored to the traditionalist vision of organizing information through “drive, folder or excel”, reflecting the resistance to change.

**Conclusions:**

The imperative need for continuous training in the use of bibliographic managers in both students and teachers is asserted, reflected as a transversal strategy throughout all academic cycles and semesters and internalized as a research policy that leads to generate a new change in the conception of researching reliable scientific sources, optimizing time, organizing information and guaranteeing quality research.

## Introduction

Research is emerging as one of the fundamental axes of university education, in synergy with training and extension, generating research professionals of the highest quality, helping to establish solid scientific, technological, cultural, social, and humanistic foundations that support the autonomous, independent, and sovereign progress of a nation. ^1^

From this point of view, the epistemological debate in the field of educational sciences is a challenge that is becoming more and more challenging, ^2^ since one of the dilemmas that the field of education is going through refers to the consciousness of the researcher, the accumulation of information, the recognition of scientific information, the use of different bibliographic managers that allow the organization of information, the axiological matrices that govern their thinking, the state of comfort, the epistemic instrumental power that anchors their paradigmatic vision of seeing, understanding and interpreting the cosmos, aspects that limit the spaces for collective reflection and the reality of a planet life in constant transformation. ^3^

Therefore, the researcher in the educational sciences, and specifically in the university context, must unravel the multiple visions and underlying ontological complexities of human environments. In this sense, knowledge as a polyvalent source is situated in the plane of subjectivity and emergent expression of reality, understanding that knowledge is the product of a recurrent and interactive dialogic linked to historical, cultural, and social elements that mark the epistemic communities in the order of the individual and collective, interpreted in man as congenital elements in his way of thinking. ^4^

Then, the man seen as a biopsychosocial-spiritual-ecological being and creator of multiple ways of thinking, leads to perennially directing, according to his geo-historical moment and his epistemic matrix; which implies thinking and rethinking reality from an emerging research future ^5^ of great epistemic value. This allows describing and discovering the complex connections and core interactions that configure a new polyvalent architecture of thought, a new paradigmatic vision, and therefore, a new social researcher, committed to the human value, the understanding of the other, the empathic capacity to understand both tacit and explicit knowledge, allowing glimpses of a new paradigm.

Therefore, universities, as a space for divergent thinking, must promote the integral formation of their students and the development of research competencies, contributing to the formation of a leading, autonomous and self-critical citizen of their academic and research process. Consequently, there is an urgent need to understand from the perception of university teachers the conceptions about the use of bibliographic managers, as an aspect that allows organizing and systematizing scientific information, recognizing that we are currently facing a knowledge society, that requires university teachers to know how to adequately select information for their research process. ^6^

In these current times of uncertainty and where the human being is immersed in a large amount of information, it is necessary for university teachers to use bibliographic managers that allow them to systematize the information, in other words, to analyze, understand and interpret those documents with scientific validity, leading to the development of informational research. This aspect is relevant for the university professor, since it allows from his educational praxis to promote the appreciation for validated scientific information in his students, originating in them the constant questioning of what they review and read.

Furthermore, the use of scientific databases connected to bibliographic managers in university spaces allows the information to be investigated to have a scientific backing, in other words, university professors must become promoters of the investigative action, providing a scientific accompaniment that allows students to obtain good sources of scientific information or scientific data that allow them to interpret and understand the phenomenon to be investigated.

Therefore, the use of bibliographic managers, such as Mendeley, Zotero, EndNote Basic, RefWorks, among others, allows the management of scientific information through the storage and organization of information, in addition to the dissemination of research, the management of citations and references, facilitating the bibliographic adaptability of research to certain standardized styles in an automatic way.

Bibliographic managers are defined as software tools that automatically collect bibliographic reference data from sources selected from various existing databases. ^7^^,^^8^ These programs also allow the management of data obtained in any of the citation styles, ^9^ being therefore tools mainly used in the field of scientific research. ^10^

These computer programs initially emerged in the 1980s, the pioneers were EndNote, Reference Manager, and ProCite. ^11^ From there, more appeared, which can be classified into three: desktop programs, which are installed on the computer and work from there; web environment programs, which store the information on a web server that can be accessed from anywhere. ^12^ And, finally, the recent social reference managers which facilitate collaborative work on reference collections. ^13^ Desktop managers are licensed and require a subscription to access their functions, as is the case of ProCite and EndNote. ^7^ Meanwhile, managers such as RefWorks and Zotero, being web-based, are the best known. As for the latter type of managers, Mendeley and Citeulike stand out. ^12^

The advantages granted by bibliographic managers are varied, ranging from the generation of citations and references in the desired style ^14^; shared use of personal libraries with other users, through the cloud ^2^; import, export and manipulation of the data and metadata. ^3^ This facilitates the process of scientific writing, improving time management, organization of sources by criteria, access to reliable database sources, correct writing of citations according to the required style. ^15^

While there are many advantages offered by this type of programs, there is still not much use at the university level, ^2^^,^^5^^,^^16^^–^^18^ by students and teachers; this is mainly due to the lack of research policies that strengthen research and information competencies among teachers and students.

In view of this, there is a need to include the teaching of bibliographic managers in the curricula and to promote their use through formative research. The proper management of bibliographic managers, as has been argued in several studies, ^16^^,^^19^ guarantees products of higher academic quality.

Under this reality, the present research sought to understand the implicit theories on the use of bibliographic managers in the organization of scientific information in university teachers in Latin America (Colombia, Ecuador and Peru). For this purpose, the general problem was formulated: What are the implicit theories on the use of bibliographic managers in the organization of scientific information in university teachers in Latin America (Colombia, Ecuador and Peru)?

## Methods

The research assumed the interpretative paradigm, since it allowed to understand from the perceptions and experiences lived by university teachers the use of bibliographic managers. In addition, the research was approached from the qualitative approach, allowing the description of the research phenomenon without emphasizing measurement, since its purpose is the interpretation of reality. ^20^ In terms of design, it assumed a flexible, dynamic, and changing design, because it is developed through sequential stages, which are derived from each other. ^1^ The method used was phenomenological, which led the researchers to immerse themselves in the consciousness of the key informant in order to understand the input category, such as the use of bibliographic managers.

The research project was reviewed by the Ethics Committee of the Universidad Nacional Experimental Francisco de Miranda and approved with code D.A.I.2022.03.015, on March 15, 2022. The committee verified that no human condition was altered, in addition to considering the informed consent of the people who were part of the study sample; likewise, the purposes of the study were notified in advance to the key informants, who were selected in an equitable manner; in addition, the protection of the participants’ data as well as their personal information was guaranteed; and finally, it was ensured that the data collected were used only for the purposes expressed in this research. Likewise, each of the teachers provided informed consent and voluntarily authorized their desire to participate in the interview, through a written format. The interviews lasted approximately 15 minutes with each key informant. Only the researchers in charge and the key informants were present during the interviews.

The setting for the study consisted of three Latin American universities, the Universidad Cooperativa de Colombia, the Universidad Internacional del Ecuador and the Universidad Nacional de Educación Enrique Guzmán y Valle in Peru. The sample consisted of 15 teachers, five from each university who were currently teaching the research subject and who were available to participate in the study. It is worth mentioning that there was a relationship prior to the study with the teachers from the country of origin, but not with the teachers from Ecuador and Colombia; the snowball technique was key for the sampling. All the participants contacted for the interview process answered the questions; none of them left the study. The semi-structured interview was used as a technique, which allowed to understand from the realities lived by university teachers the use of bibliographic managers, also allowed the processing of data by collecting the opinions of the sample participating in the study. The instrument used was the interview script, which was composed of eight open-ended questions, arising from the objectives of the study and from the categories proposed a priori.

The semi-structured interviews were based on the interview script referred to the topic of the study and were conducted using Google Meet as an instrument that accurately recorded the information provided by the key informants. It was not necessary to repeat any interview, and once the information was obtained, it was transcribed verbatim for the information analysis process, which included the study of microanalysis or content analysis line by line, word by word, of each information protocol. Along with the microanalysis or content analysis, the method of constant comparison between each of the dimensions and properties was carried out simultaneously, achieving consistency. Likewise, the first process of internal reliability was achieved, since the key informants confirmed that it was the product of the interaction between them and the researchers.

The interviews were conducted during the month of July 2022, where we coordinated first with each teacher, according to the availability of each one of them to collaborate with the research. The study was based on four cyclical-recursive moments, the first was the natural attitude, where information was obtained from the key informants as a result of the interviews, for transcription, analysis and coding. ^21^ The second moment allowed the epoche - eidetic reduction, consisting of the analysis of each interview to extract the phrases, words, or attributes that stood out in the process. The third moment originated the epoche-transcendental reduction, analyzing each attribute and their consistency in each interview analysis, allowing the emergence of attributes, subcategories, and emerging categories. The fourth moment revealed the constitution of the study, integrating in a semantic network the categories, subcategories and attributes that emerged in the research from a holistic, systemic and integrating vision. For the analysis of the data, the ATLAS.ti version 22.0.0 program, with license ID: L-A44-93E, was used, which allowed the introduction of the data, originating the interrelationships between each one, for their subsequent interpretation of the reality studied. Similarly, the same process can be carried out with the free software NVivo.

## Results

The results of the research are presented below, according to the moments established for the same.

### First moment of the natural attitude

Once the information was obtained, all the interviews were transcribed verbatim, ^26^ achieving the first process of internal reliability, since the key informants confirmed that the information was the product of the interaction between them and the researchers. Once all the data had been transcribed, we proceeded to the content analysis line by line, words or phrases, originating the microanalysis, as expressed by. ^22^

It should be noted that the four researchers coded the teacher informants to protect their personal data. The name Doc. refers to teachers, then the country of origin and teacher number. For teachers from Peru, the coding was determined as follows: Doc-Peru-No. 1, Doc -Peru-No. 2, Doc -Peru-No. 3, Doc -Peru-No. 4 and Doc -Peru-No. 5. For teachers from Ecuador: Doc -Ecuador-N° 1, Doc -Ecuador-N° 2, Doc -Ecuador-N° 3, Doc -Ecuador-N° 4, Doc -Ecuador-N° 5. And, for teachers from Colombia: Doc -Colombia-N° 1, Doc -Colombia-N° 2, Doc -Colombia-N° 3, Doc -Colombia-N° 4, Doc -Colombia-N° 5.

In the same vein ^22^ microanalysis in the interview “requires careful and often even meticulous examination and interpretation of data” (p.64). This analytical process in the research became a methodical element in the line-by-line investigation of the data, until the semantic units that emerged from reality were deciphered. From this perspective, microanalysis in the research involves transcribing verbatim all the interviews conducted with key informants, underlining with a specific color the most important phrases or data, with the function of making special emphasis, emerging the semantic units for their subsequent interpretation in scientific language.

### The second moment, epoche-eidetic reduction

This process was carried out according to each stated research objective and according to the characteristics of the key informants. Therefore, the following is an outline of this process ( Table 1).

**Table 1.** Eidetic reduction of university teachers.

Natural thematic unit: assessment of the organization of information as an investigative process						
Questions	Doc-Ecuador-N° 1	Doc-Ecuador-N° 2	Doc-Ecuador-N° 3	Doc-Ecuador-N° 4	Doc-Ecuador-N° 5	Semantic unit
What aspects do you consider to be involved in the organization of information in research?	The speed and ease of organizing the information in general, the cost of learning are key elements.	Availability: once we are clear about the topic to be investigated and the necessary information, we must investigate whether there is information from secondary sources on the subject. Whether in books, magazines, digital databases, etc. The second aspect to consider is the year of such information, since it is recommended that it be as recent as possible, a maximum of 5 years. Another aspect to consider is the line of research, and that it is related to the one of the researchers.	In my opinion, the organization of the information will depend on the type of approach to be used in the research. In my experience, using a quantitative approach, the data for the methodology must be organized and ready for use. As a second point, in both approaches, the state of the art must be organized according to the discussion of the results. Frequently, there is research whose discussion (comparing results with previous work) is not related to the references used in the literature review section.	It is very important to systematize the theoretical and epistemological foundations that will in one way or another support our research, this systematization is supported by certain resources such as bibliographic organization managers.	The researcher's access to different databases and information is important, as well as the proposed methodology to be applied and, obviously, the researcher's experience and knowledge.	Time optimization Availability of information Research approach State of the art Systematize theoretical foundations Bibliographic managers Database access Experience and knowledge
When conducting your research, do you consider the organization of the information to be important? Why?	Yes, because we have organized the ideas and generated a potential of relevant aspects that are missing in the subject and that could generate other research topics to detect opportunities for improvement.	Of course, since the organization allows to be efficient in the management of the time spent on reading and research. By organizing information by author, year, subject, we can filter specifically the type of material we want to research for our study and be faster in organizing bibliographic references.	Of course, having information in order will facilitate the correct drafting of the article. It takes a long time to search for information from previous works, and not having this information in order could take more time to organize it in order to proceed with the elaboration of the research.	It is important and difficult to organize the information, since it helps us to delimit it and to be clear about which authors we are going to work on the new discoveries and also to discard information that does not contribute to the research process. That is why it is very significant to organize the information, update the sources, consult the scientific validity and update it.	Research is a continuous process. When we start working on a topic, in order not to duplicate efforts, we have to take over from the latest research on the topic we are going to investigate and for this we must have a previous organization.	Organization of information Generation of new research Time optimization Facilitates writing Thematic delimitation Scientific validity Updated sources
How is the organization of information processed in the research?	I use a matrix where I feed adding paper aligned to the theme. Mendeley helps to submit references and have a fixed database. The initial cost of learning, which takes time to learn and make useful, must be considered. Working with Mendeley in a team is complicated if not everyone is familiar with it.	Usually, through programs or applications that automate the creation of bibliographic references used in research papers.	Through specific software. My preferences are towards Stata, Rstudio, Mendeley, Zotero and Endnote. I prefer to use Mendeley because, besides being a free software, it is connected with SCOPUS and gives me a better help in terms of placing references in my work.	I start from conceptual axes that help me to organize and be clear about the theories I am going to investigate and develop the research from headings that arise from the conceptual axes.	I do not work with bibliographic managers due to lack of knowledge and that the research department should constantly train teachers and have access to the tools.	Matrix Reference Programs Mendeley Conceptual axes STATA RStudio Zotero EndNote

*Note.* Interviews to university professors.

### The third moment, called epoche-transcendental reduction.

At this point, the results are integrated according to the categories, subcategories, attributes and transcendental ideas that emerged from the research process based on the interviews conducted with university teachers from Peru, Ecuador and Colombia ( Table 2).

**Table 2.** At this point, the results are integrated according to the categories, subcategories, attributes and transcendental ideas that emerged from the research process based on the interviews conducted with university teachers in teachers in Peru, Ecuador and Colombia.

Category	Subcategory	Attributes or codes	Important aspects that emerge	Transcendental idea	Interpretation
Bibliographic managers	Assessment of the organization of information	Literature on the subject Systematize theoretical foundations Thematic clarity Time optimization Information management and organization Order Facilitates writing Provides citation and bibliography Useful sources Updated sources Copyright Scientific validity Identification of original authors Mendeley Zotero EndNote Referencing programs	Topic identification Time optimization References Scientific validity Bibliographic managers	The perspective of university professors emphasizes the process of organizing the information from the establishment of the topic, being for them the key and initial process. In addition, they consider that organization is a process that allows an optimal use of time, because it facilitates several activities of the research process; and it grants scientific validity to the research work, since it guarantees reliable and current sources. This is what happens, according to them, with bibliographic managers as tools for organizing information.	The holistic vision of university teachers lies in the importance they give to the identification of the subject and the usefulness of reliable sources in the process of organizing information. Teachers understand the importance of the information organization process and associate it with the use of tools such as bibliographic managers and databases. They understand that this process facilitates the following phases of the research and establishes the bases that guarantee the quality of the study to be developed.
	Conceptions on the use of bibliographic managers	Lack of knowledge of managers. Self-taught. Mendeley. Zotero. Organize information. Digital era.	Manager training Bibliographic managers		
	Bibliographic managers as a technological tool	Variety of sources. Support of the sources consulted. Certification and credibility.	Scientific sources		
Organization of information	Digital literacy - scientific research process	Manages updated thematic inputs. Guarantees an orderly work. Reliable organizer. Thematic relevance. Review of the topic and subtopics.	Manages reliable information ICT and research Reliable sources	From the teachers' perspective, they point out that this process facilitates and manages resources for information, allows the creation of a database whose source is reliable as well as guarantees the quality of the research. They frequently use these tools because it gives them greater security in the construction of information.	By understanding the teachers' perspective in an integrated manner, the use of these bibliographic managers improves the organization of information through the establishment of a reliable database, the quality of information and the management of resources that will make relevant research possible.
	Research process - organization of scientific information	Organize and select sources of information. Database. Citation.	Database		
	Technological tool for the management of scientific information	Facilitates the organization of information. Guarantees the quality and accuracy of the information. Teachers and students involved in research.	Quality of information Reliable information Academic and scientific community		

Note. Categories of the study based on the analysis of data on teachers in Peru, Ecuador and Colombia.

### Interpretation of transcendental reduction closure in teachers from Peru, Ecuador and Colombia

When interpreting the data from teachers regarding the use of bibliographic managers, it is emphasized that teachers currently use different technological platforms for research, so the selection of information is essential and they must have skills that allow them to use indicators of quality and reliability of the information. However, even when they recognize the importance of the use of bibliographic managers and a culture of searching in online libraries, they do not seem to give importance or applicability to the use of bibliographic managers in their academic-scientific activities, aspects that are interpreted as negative, since the traditional way of processing and organizing information through “folder, drive or Excel sheet” is still in force, limiting the use of these scientific tools to its minimum expression.

In this regard, it should be understood that currently the information society is voracious, every day the human being is faced with a large amount of data that make it diverse, prolific and varied. Therefore, the use of bibliographic managers in academic spaces and from the conception of the university teacher, should become a tool that transcends the cognitive-informational level and takes possession of the pragmatic and everyday academic activities, contributing to the educational formation of students.

From this perspective, the use of bibliographic managers in higher education should not only be limited to research processes, but should permeate all academic-curricular activities, being reflected as an educational policy in university activities. In this sense, a priority for researchers is the knowledge on the use of technological tools, and to have easy access to scientific information; it is necessary that both teachers and students know and have access to relevant information for research work, making constant use of bibliographic managers. It is necessary to have scientific documents available for open consultation to enrich scientific work and make it much more effective.

### Fourth moment, constitution

**Figure 1.**
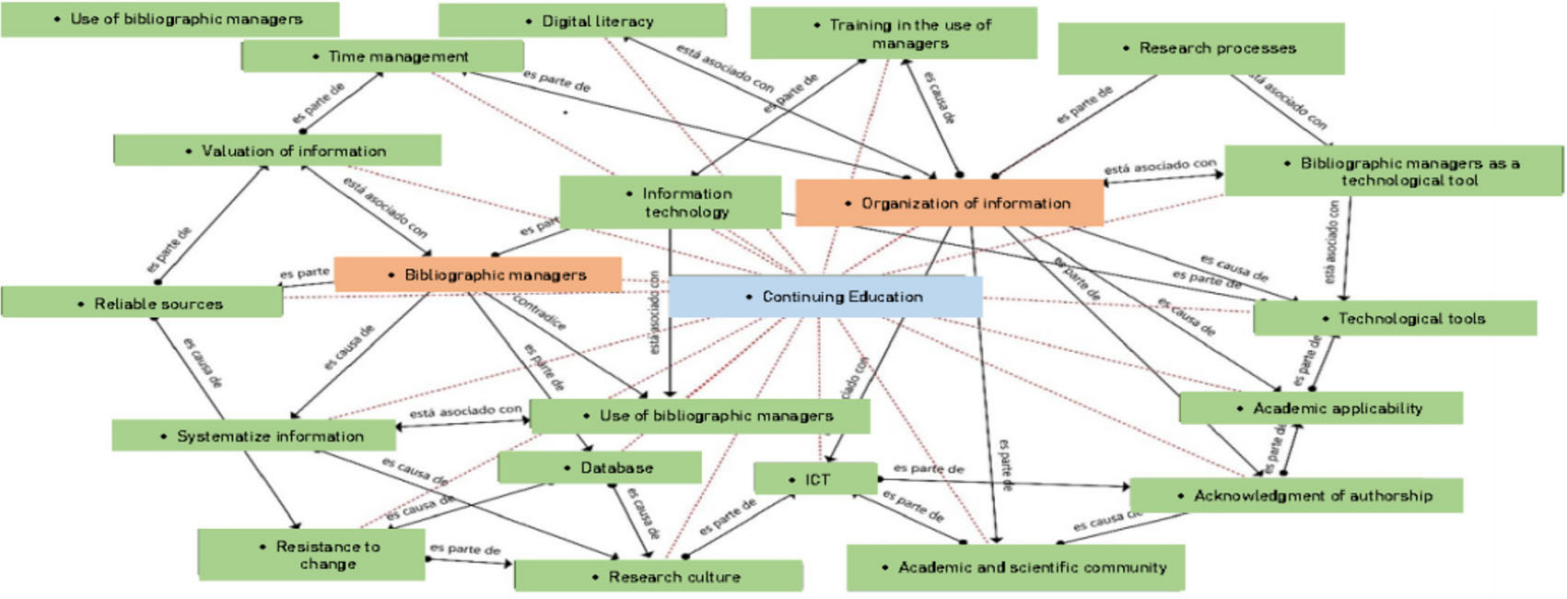
Semantic network integrating bibliographic managers, information organization, continuing education. Note. Categories of the study based on the analysis of data on teachers in Peru, Ecuador and Colombia. Es parte de: is part of; Es causa de: is the cause of; Está asociado con: is associated with; Contradice: contradicts.

At this point we present the semantic networks of how the categories, subcategories, attributes, emerging categories, transcendental ideas are integrated from an integrative, systemic and holistic view of the study phenomenon, based on the interviews conducted with university teachers from Peru, Ecuador and Colombia, as shown in Figure 1. ^27^

### Interpretation of the integrating network bibliographic managers, organization of information, continuing education

The following are the results of the research, which show the imperative need for continuing education in the use of bibliographic managers in both students and teachers, reflected as a transversal strategy throughout all academic cycles and semesters and internalized as a research policy that leads to generate a new change in the conception of researching reliable scientific sources, optimizing time, organizing information and ensuring quality research.

Under this perspective, the use of bibliographic managers in university academic spaces focuses the bases for continuing education, understanding that at the present time a large amount of data and information emerges as a result of information and communication technologies, which requires both students and teachers to verify the sources consulted. Under this vision, the research processes are assumed from the freedom of thought, the constant questioning of reality, the quality of the information, understanding that the evaluation of the information constitutes permanent processes on the research praxis.

Under these axioms, the use of bibliographic managers in Higher Education should be reflected as a tool of open access to the university community and permeate the curriculum, that is, it should be reflected in each course or subject, academic and scientific activity as a daily tool that leads to generate critical and reflective citizens of the researched reality, reflecting its use in the academic applicability.

In the same vein, the use of bibliographic managers cannot be reflected only in research activities, but rather, its usefulness must be understood in every space where man generates knowledge, that is, from the different academic activities performed by both students and teachers. In addition, university teachers should appropriate this tool as a ramified axis in their educational praxis that leads to integrate and include more students about the benefits and usefulness of this tool in their academic studies, in their personal and professional lives. In this respect, university learning spaces should promote the integration and inclusion of bibliographic managers as a daily praxis that invites the educational actors involved in processes of constant reflection on their reality, providing alternative solutions that contribute to the common good.

In these times, when there is an accumulation of data products of the information society, it is necessary to value the information as an essential point to resort to validated scientific sources, leading both teachers and students to systematize information, to obtain valuable data for the interpretation according to the researched topic, to order it properly and above all, to manage time, as a process to optimize the quality of the information. Under this vision, all information reviewed must meet reliable standards that lead to generate added value within a knowledge society that demands reliable sources of the information produced.

Therefore, it must be understood that information technologies are generating an endless amount of information data every day, which makes it necessary for people to value the information they are researching, obtaining reliable sources and making use of bibliographic managers as a way to organize it and optimize their academic productivity.

Therefore, university education systems must keep pace with social changes, in line with the needs of the context, of man himself and his ecosystem, in order to generate useful knowledge for life. In this way, the use of bibliographic managers should not be reflected as a content of a course or subject, but rather as a transversal strategy of academic training that leads to its pragmatic and practical use, internalizing it as an essential tool in the university academic life of all the people involved in the process.

It is therefore urgent, from the institutional management of higher education, academic policies that lead to the appropriation and empowerment of the use of bibliographic managers by all the educational actors in the process, reflected as continuing education processes on the praxis. In these times of change and accelerated information, the valuation of information is urgent, and there is a need for student-teachers who can interpret reality, question, and generate new contextualized knowledge, generating a new institutional culture in universities. It is necessary that the academic processes are in line with the changes generated by the globalizing world, in order to have an organization at the forefront and not anchored to the traditional vision. This also implies the rethinking of a new citizen, not only committed to the organization, but also to his/her social environment and his/her own life, since his/her actions are reflected in his/her social context and become models of inspiration for others.

Therefore, it is asserted that bibliographic managers become an essential tool in the processes and educational formation of the new citizen that society deserves, since it is of vital importance within the daily educational university work, allowing to reduce the uncertainty generated by the information society.

## Discussion

This changing and complex world that constantly generates large-scale information data, has also generated Big data and constant changes in the mental structures of human beings for the process of organizing information and in this field the support provided by bibliographic managers is invaluable, as stated by the Ecuadorian researchers, ^2^ who highlight the benefits of such bibliographic managers in terms of organization, systematization and conceptualization of the information. On the other hand, Cuban authors ^18^ draw attention to the usefulness of managers for young researchers with difficulties in the use of the Apa-Vancouver.

The use of bibliographic managers makes it possible to optimize the time available to the researcher for accessing and organizing information, ^23^ which is extremely important in an era of large distances between the time available to the researcher and the volume of information that can be accessed. In Costa Rica, the researchers ^24^ state the relevance of the use of managers as research tools.

The researcher’s challenge is to provide itself with technological tools that allow it to optimize the results and quality of its research. Other benefi-ts provided by bibliographic managers are the socialization of research results and collaborative work, as Ref. 6 point out.

In reference to the conceptions about the use of bibliographic managers in the organization of scientific information in teachers, the findings establish that there is an urgent need to incorporate the use of bibliographic managers as a skill in university students and teachers. This can be done through information literacy programs, as pointed out by Ref. 23 or incorporated into the syllabus of a subject of the research area. While it is true that managers are research tools and play a key role in developing students’ research skills, ^24^ it is not only the curricular research experiences that must guarantee their use, but also the entire curriculum, since promoting, their use in isolation from other curricular experiences would not be sufficient to generate an internalization of their use. ^2^

Therefore, it is necessary to conceive it as a transversal strategy during the period of higher education, and this is possible if it is established as a guideline or institutional policy for the elaboration of academic products; this guarantees the execution of an adequate organization of information ^4^^,^^17^^,^^25^ the optimization of time, and above all, the quality of the research or academic product. This last aspect becomes relevant when it becomes evident that the main difficulties of students is the search for sources and the citation and referencing of them ^2^^,^^18^; which explains why bibliographic managers would become indispensable tools for the student body. ^6^^,^^8^^,^^11^^,^^17^

The implementation of the use of bibliographic managers as an institutional guideline not only leads to the obvious development of students’ research competence. This was demonstrated in the study by Ref. 23 in which it was found that users of bibliographic managers spend a tenth of the time to make a citation than those who do not know how to use this tool. In addition, the use of these programs is reflected in an improvement in academic performance in general, ^4^ which would help us to achieve the long-awaited educational quality.

## Conclusion

According to the general objective, to understand the implicit theories about the use of bibliographic managers in the organization of scientific information, in university teachers in Latin America (Colombia, Ecuador and Peru), it is asserted that even when it is recognized from the cognitive conception about the concept of bibliographic managers, its applicability or use, it seems not to be so effective in scientific academic spaces and activities, because teachers are still anchored to the traditionalist vision of organizing information through “drive, folder or excel”, reflecting the resistance to change. The use of bibliographic managers requires formative spaces, instruction, and opportunities that strengthen professional competence in the research process, in a way that is reflected in the performance as evidence of the educational quality that is created.

## Data Availability

Figshare: Interviews.docx, https://doi.org/10.6084/m9.figshare.25274098. ^26^ This project contains the following underlying data:
• Interviews.docx Figshare: Figure 1.jpg, https://doi.org/10.6084/m9.figshare.25016429. ^27^ This project contains the following extended data:
•
Figure 1. jpg Figshare: COREQ Checklist for ‘Conceptions of bibliographic managers in university teachers. An approach to the reality in Latin American’, https://doi.org/10.6084/m9.figshare.25016453. ^28^ Data are available under the terms of the Creative Commons Attribution 4.0 International license (CC-BY 4.0).
