# Supplementary material for: Conceptions of bibliographic managers in university teachers. An approach in Latin American
Source: F1000Res. 2024 Mar 21;13:207. [Version 1] doi: 10.12688/f1000research.143691.1 (PMC11109577; doi:10.12688/f1000research.143691.1)
Supplement: Peer review history

# Reviewer report: Reviewer response for version 1

**DOI:** 10.5256/f1000research.157378.r262224

**Published:** 2024-05-09

**Authors:** Luis Marcelo Olivos Jimenez

**Affiliations:** 1 Ancash, Universidad César Vallejo (Ringgold ID: 488352), Chimbote, Ancash, Peru

The article approaches in an integral way allowing to understand the reality of the problem from the Latin American countries in attention to the conception of university teachers about bibliographic managers. The study was approached from a qualitative approach, leading to understand the lived realities from the experience. The technique and the instrument were appropriate to understand the phenomenon of the study. The criteria for the selection of the study sample and the informed consent are clearly presented. The results present important elements of interpretation according to the context of higher education. However, the study could be further improved by considering some recommendations. First, it would be beneficial to go deeper into the reality of the problem by detailing the specific problems. Second, the hypotheses and conclusions of the study should be strengthened.

Is the work clearly and accurately presented and does it cite the current literature?

Yes

If applicable, is the statistical analysis and its interpretation appropriate?

Yes

Are all the source data underlying the results available to ensure full reproducibility?

Yes

Is the study design appropriate and is the work technically sound?

Yes

Are the conclusions drawn adequately supported by the results?

Yes

Are sufficient details of methods and analysis provided to allow replication by others?

Yes

Reviewer Expertise:

University teaching, methodology in scientific research, technology and innovation in public management, digital skills.

I confirm that I have read this submission and believe that I have an appropriate level of expertise to confirm that it is of an acceptable scientific standard.

# Reviewer report: Reviewer response for version 1

**DOI:** 10.5256/f1000research.157378.r262223

**Published:** 2024-05-09

**Authors:** Eduardo Jesús Garces Rosendo

**Affiliations:** 1 Universidad Tecnologica del Peru, Lima District, Lima Region, Peru

The article exposes the conceptions that university teachers have about bibliographic managers. The research has a theoretical support from updated sources that allow a better explanation of the reality in the Latin context. The qualitative approach used was the most adequate for the research. The method and analysis carried out to meet the objective of the study have been specifically detailed. The data processing allowed the understanding of the study phenomenon, which led to detailed and focused conclusions on the established purposes. Finally, the source data have been appended to Figshare, ensuring the transparency of all data collected and their reproducibility. The study makes an essential contribution to university teaching. It is recommended that the authors: (a) Clearly detail the specific problems of the study. (b) Clearly specify the key informants, detailing the sample selection criteria. (c) Verify that the specific problems are addressed in the results.

Is the work clearly and accurately presented and does it cite the current literature?

Yes

If applicable, is the statistical analysis and its interpretation appropriate?

Yes

Are all the source data underlying the results available to ensure full reproducibility?

Yes

Is the study design appropriate and is the work technically sound?

Yes

Are the conclusions drawn adequately supported by the results?

Yes

Are sufficient details of methods and analysis provided to allow replication by others?

Yes

Reviewer Expertise:

educational technology, teacher training, research

I confirm that I have read this submission and believe that I have an appropriate level of expertise to confirm that it is of an acceptable scientific standard.
